# Autophagy: An Essential Degradation Program for Cellular Homeostasis and Life

**DOI:** 10.3390/cells7120278

**Published:** 2018-12-19

**Authors:** Yoomi Chun, Joungmok Kim

**Affiliations:** Department of Oral Biochemistry and Molecular Biology, School of Dentistry, Kyung Hee University, Seoul 02447, Korea; ychun@khu.ac.kr

**Keywords:** autophagy, autophagy adaptor, AMPK-mTORC1-ULK1 triad, PIK3C3/VPS34, regulatory modifications

## Abstract

Autophagy is a lysosome-dependent cellular degradation program that responds to a variety of environmental and cellular stresses. It is an evolutionarily well-conserved and essential pathway to maintain cellular homeostasis, therefore, dysfunction of autophagy is closely associated with a wide spectrum of human pathophysiological conditions including cancers and neurodegenerative diseases. The discovery and characterization of the kingdom of autophagy proteins have uncovered the molecular basis of the autophagy process. In addition, recent advances on the various post-translational modifications of autophagy proteins have shed light on the multiple layers of autophagy regulatory mechanisms, and provide novel therapeutic targets for the treatment of the diseases.

Autophagy is a fundamental cellular homeostasis program that deals with harmful or surplus cellular contents such as protein aggregates, dysfunctional/long-lived organelles, intracellular pathogens, and storage nutrients (glycogen and lipid droplets) [1,2,3,4]. Autophagy degrades these cytoplasmic components by delivering them into the lysosomes (or vacuoles in yeast). Of note, autophagy functions as a recycling and fueling program to provide sources of energy or building blocks for the synthesis of macromolecules. 

Autophagy in mammals can be categorized into macroautophagy and chaperone-mediated autophagy (CMA), depending on how the autophagic substrates move to the lysosome. Microautophagy has been reported in yeast, where the destructive cargoes are directly engulfed by a yeast lysosomal compartment, the vacuole. CMA delivers the destructive substrates, mostly cytosolic protein aggregates, into the lysosome with chaperons Hsc70 (a heat shock cognate protein of 70 kDa). The Hsc70-guided CMA cargo complex is recognized by the lysosomal membrane receptor LAMP-2A (lysosome-associated membrane protein type 2A). CMA cargo proteins have a consensus CMA-targeting motif (KFERQ) for Hsc70 interaction. The CMA-targeting motif, in general, is buried or masked, but is exposed to form a complex with Hsc70 when the CMA signaling occurs [5]. Macroautophagy (hereafter referred to as autophagy) uses a unique transport vesicle with a double-membrane structure, an autophagosome, to deliver its destructive cargos to the lysosome [6]. The autophagosome then fuses with the lysosome to form the autolysosome, where the cargos are eventually degraded by lyososomal acid hydrolases. Extensive genetic and biochemical studies in yeast and fly have provided a milestone of mechanistic insight into autophagy, notably leading to the discovery of the autophagy-related gene (ATGs) family [7]. ATG proteins and a variety of non-ATG proteins, for example, VPS (vascular protein sorting) proteins, Rab small GTPases, and SNARE proteins, consist of an autophagy machinery network for autophagosome biogenesis, autophagosome-lysosome fusion (autolysosome), and autophagic degradation and recycling. Excellent reviews demonstrating the molecular details of autophagy machinery and autophagosome/autolysosome biogenesis can also be found elsewhere [6,7,8,9,10]. 

## 1. Key Autophagy Machinery Proteins in Autophagosome Biogenesis

**Figure 1:**
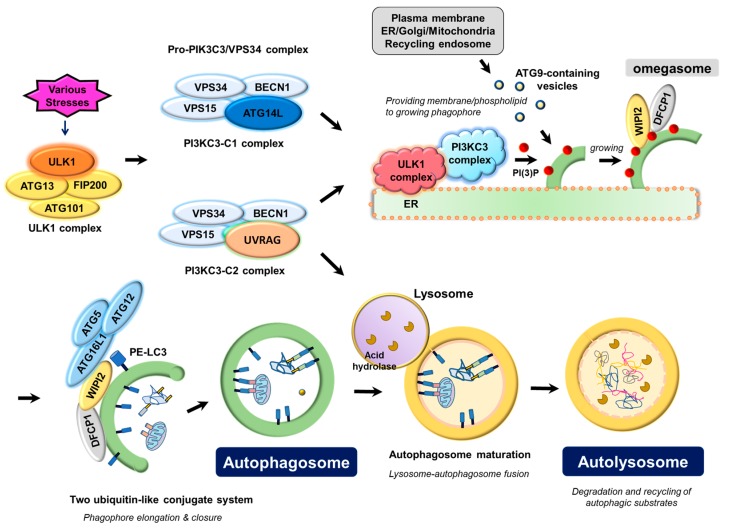
Molecular mechanism underlying autophagosome biogenesis by the coordinated actions of ATG proteins. Upon various stresses, the ULK1 complex, consisting of the catalytic subunit ULK1 protein kinase and its associated-regulatory subunits such as ATG13, FIP200, and ATG101, triggers nucleation of the phagophore by phosphorylating and activating the pro-autophagy PIK3C3/VPS34 lipid kinase complex containing either ATG14L (PI3KC3-C1) or UVRAG (PI3KC3-C2), which in turn marks a distinct ER membrane with its phospholipid product, PI(3)P, to form omegasome. PI(3)P on omegasomes then recruits the PI(3)P effector proteins, WIPI2 (WD repeat domain phosphoinositide-interacting protein 2) and DFCP1 (zinc-finger FYVE domain-containing protein 1). WIPI2 and DFCP1 function to gather two ubiquitin-like conjugate complexes, ATG12-ATG5-ATG16L1 and phosphatidylethanolamine (PE)-conjugated LC3 (LC3-II) for elongation and closure of the phagophore membrane. Plasma membrane, mitochondria, recycling endosomes, or Golgi complex may contribute to the elongation of the autophagosomal membrane by providing part of their membrane layers via ATG9. Closure of the phagophore membrane gives rise to a double-membrane bounded vesicle called the autophagosome, which matures and finally fuses with the lysosome to form the autolysosome. Acidic hydrolases in the lysosome degrade the autophagic cargo, and the degradative products are recycled to cope with the stresses that the cells encounter.

Autophagosome biogenesis is carried out by the coordinated actions of autophagy machinery proteins, of which the ULK1 complex and pro-autophagy PIK3C3/VPS34 complex function as most upstream and key regulators in autophagosome biogenesis and autophagosomal membrane nucleation. In addition, two ubiquitin-like conjugate systems (ATG12-ATG5-ATG16L1, LC3-II) and ATG9 are necessary for phagophore (preautophagosomal structure) membrane elongation and closure for autophagosome formation (Figure 1).

### 1.1. ULK Complex

Lines of evidence have demonstrated that the ubiquitously expressed protein kinases ULK1 and ULK2 have the most upstream and central position to trigger the autophagy program [10,11]. ULK1/2 form a complex with FIP200, ATG13, and ATG101, of which ATG13 and FIP200 have been reported to stabilize ULK1, increase its kinase activity, and play a role in translocation of the ULK1 complex from the cytosol to the omegasome, a unique membrane structure initiating the phagophore formation [12,13]. Similar to the domain structure of ATG1 (a yeast homologue for mammalian ULK1), ULK1 has an N-terminal protein kinase domain, a central intrinsically disordered region (IDR) that is a target for various phosphorylations, a LC3-interacting region (LIR), and C-terminal early autophagy targeting and tethering (EAT) domain that interacts with ATG13 [14]. Interestingly, the formation of the ULK complex is not regulated by nutrient status [15], whereas the ATG1 complex, a ULK1 orthologue in yeast, is known to be sensitive to amino acids [16,17], suggesting the different regulatory mechanisms between yeast and mammals.

Although ULK1/2, in general, have redundant functions, distinct roles between ULK1 and ULK2 have also been reported, especially in lipid metabolism [18]. Additionally, ULK1/2 appear to have additional unique functions that are not observed in the inhibition of other ATG proteins such as ATG5 and ATG7 [19]. For instance, although neuronal degeneration was similarly observed in brain-specific ULK1/2, ATG5, and ATG7 knockout mice [20,21], the ULK1/2 knockout mice did not show the accumulation of the p62/SQSTM1-ubiquitin double-positive inclusion body and abnormal membrane structures in the neurons [22]. It has been demonstrated that neuronal defects shown in these ULK1/2 knockout mice resulted from the dysregulation of the unfolded protein response in neurons [22]. A recent report also showed that, in parallel with the classical role in autophagy induction, ULK1/2 functioned in the maintenance of intracellular energy and redox balance by reprograming metabolic flow to the pentose phosphate pathway, providing NADPH for ROS scavenging while sustaining overall glucose catabolism in response to nutrient starvation [23].

The ULK complex is mostly cytosolic, but a fraction of the ULK complex can also be observed in the endosomes, mitochondria, and the ER [13]. Upon amino acid starvation, the ULK complex translocates to the regions that are in close proximity to the ER-membrane contact sites, for example, ER-mitochondria [24] or ATG9 positive autophagy-specific ER exit sites [25]. The C-terminus of the ULK1 EAT domain as well as the N-terminal basic patch of the ATG13 on the ULK1 complex play an important role in the complex anchoring to the omegasome [26]. In addition, phosphatidylethanolamine (PE)-conjugated LC3 (LC3-II) on the phagophore binds to ULK1 and ATG13 to further increase their retention at the phagophore [27,28]. A recent study has documented that the Rab1a effector C9orf72 binds to ULK1 complex to promote Rab1a-dependent trafficking of the ULK1 complex to the phagophore [29]. Accordingly, the reduction of C9orf72 expression in cell lines and primary neurons attenuated autophagy and caused the accumulation of p62/SQSTM1-positive puncta, which may underlie the neuro-pathophysiology frequently observed in amyotrophic lateral sclerosis and frontotemporal dementia (C9ALS/FTD) patients. Once the phagophore membrane has established growth, the ULK complex is recycled to the cytoplasm [26]. 

### 1.2. PIK3C3/VPS34 Complex

The class III phosphatidylinositol 3-kinase (PIK3C3/VPS34) phosphorylates phosphatidylinositol (PI) on the endosomal membrane to generate PI-3-monophosphate, PI(3)P, a key membrane marker for both intracellular trafficking and autophagosome formation [30]. PI(3)P functions to recruit proteins containing the PI(3)P-binding FYVE or PX domain, which modulate membrane architecture [31]. PIK3C3/VPS34 interacts with various proteins to form multiple complexes that are responsible for numerous cellular functions such as the multi-vesicular body pathway, retrograde trafficking from endosomes to the Golgi, and phagosome maturation as well as autophagy [32]. For autophagy, an ATG protein, ATG14L/Barkor, guides the PIK3C3/VPS34 complex to the phagophore initiation site to mark the membrane with PI(3)P for the recruitment of a set of downstream autophagy machinery, occurring just downstream of ULK1 [33]. 

PIK3C3/VPS34 forms at least two distinct complexes, PI3KC3–C1 and PI3KC3–C2, which are required for different stages of the autophagy process [34,35]. The core complex in both PIK3C3-C1 and -C2 contains the catalytic subunit VPS34, the pseudokinase VPS15/p150 (PIK3R4), and BECN1 (Beclin 1, a mammalian homolog of yeast ATG6). Depending on the subcellular context, this core complex binds to either ATG14/Barkor or UVRAG (UV-radiation resistance-associated gene protein) in a mutually exclusive manner defining the PI3K3-C1 and -C2, respectively. The UVRAG-containing PI3K3-C2 complex functions not only in autophagy, especially for autophagosome maturation and autolysosomal tubulation [36,37], but also in endosome trafficking and multi-vesicular body formation [32]. UVRAG regulates autophagosome maturation by binding to the HOPS (homotypic fusion and vacuole protein sorting) complex, which stimulates lysosomal fusion with the autophagosome [38]. This interaction between the UVRAG-containing PI3K3-C2 complex and HOPS complex is antagonized by RUBICON (RUN domain Beclin 1-interacting and cysteine-rich containing protein) [39]. In addition, RUBICON suppresses the lipid kinase activity of the PI3K3-C2 complex [40]. In the case of the PI3K3-C1 complex, it appears to be more specific for autophagy, especially for autophagosome nucleation. ATG14L/Barkor devotes PI3KC3-C1 to function as an early upstream regulator for autophagosome formation by directing the complex to phagophore initiation sites. Many efforts have shown that the N-terminal cysteine-rich domain [41] and the C-terminal amphipathic helix BATS (Barkor/ATG14L autophagosome targeting sequence) domain of ATG14L/Barkor [42] are important for the starvation-induced translocation of PI3K3-C1 to the phagophore initiation sites. Extensive molecular imaging and biochemical analysis have found that the SNARE protein syntaxin 17 (STX17) on ER recruits ATG14L/Barkor to ER-mitochondria contact sites to drive autophagosome biogenesis in a starvation-dependent manner [24]. Importantly, several ER-organelle membrane contact sites are known to function as platforms for autophagosome formation [43]. ATG14L/Barkor plays an important role in targeting the pro-autophagy PIK3C3/VPS34 complex on ER-mitochondria [24] and ER-Golgi intermediate compartment [44]. In the ER-plasma membrane contact sites, which are enhanced by starvation, an extended synaptotagmis (E-syt) recruits the PIK3C3/VPS34 complex for local PI(3)P generation [45]. Similarly, a recent report has shown that a multi-membrane spanning ER protein, VMP1, can recruit the PIK3C3/VPS34 complex to various ER-membrane contact sites, leading to phagophore elongation [46].

There are several additional PI3KC3-C1 (also for PI3KC3-C2) complex associating proteins that regulate the complex activity and targeting on phagophore initiation sites. Similar to the role of the yeast ATG38 [47], NRBF2 (nuclear receptor binding factor 2) in mammals has been shown to specifically bind to PI3K3-C1 through VPS15/p150 and ATG14L/Barkor for the complex assembly and subsequent autophagy induction [48,49]. However, the role of NRBF2 on autophagy remains to be further examined because it has been reported that NRBF2 suppresses autophagy by modulating ATG14L/Barkor-containing PI3KC3-C1 complex architecture through BECN1 interaction, which inhibits the complex activity to reduce the intracellular PI(3)P level [50]. The anti-apoptotic factor Bcl-2 also binds to the BH3 (Bcl-2 homology domain 3) domain of BECN1, which overlaps with NRBF2 binding site [51]. Bcl-2 binding to BECN1 inhibits VPS34 kinase activity and suppresses autophagy [52]. Interestingly, the interaction between Bcl-2 and BECN1 is regulated by nutrient status [53]. Starvation induces c-Jun N-terminal protein kinase 1 (JNK1)-dependent multiple phosphorylations on Bcl-2 to release Bcl-2 from BECN1, thereby, activating autophagy. A fraction of the PIK3C3/VPS34 complex has also been shown to localize onto the cytoskeleton via an association between BECN1-interacting AMBRA1 and microtubules, which is disrupted upon starvation to allow translocation into the omegasomes [54]. VMP1 binds to BECN1 to help the translocation of the complex into punctate ER-membrane contact sites [46] and VMP1-BECN1 binding antagonizes Bcl-2 interaction with BECN1 [55]. A recent study has documented that PAQR3 (progestin and adipoQ receptor 3) specifically promotes ATG14L/Barkor-containing PI3KC3-C1 complex formation as a scaffold protein, leading to the complex activation [56]. PAQR3 appears to connect the PI3KC3-C1 complex with cellular energy status via its own phosphorylation by AMPK [56].

### 1.3. ATG12-ATG5-ATG16 Complex and LC3-Phospholipid Conjugate

Two ubiquitin-like conjugate proteins, ATG12-ATG5 and LC3 (microtubule-associated proteins 1A/1B Light Chain 3), also play an important role in autophagosome biogenesis in terms of vesicular structure formation [57,58]. The ATG12-ATG5 system includes ATG12, ATG7 (E1-like), ATG10 (E2-like), ATG5, and ATG16L1 [59]. The resulting ATG12-ATG5-ATG16L1 conjugate plays a role in phagophore membrane elongation and closure by recruiting LC3 on the phagophore membrane and promoting LC3 processing. LC3 (ATG8 in yeast) is first cleaved by the cysteine protease ATG4. The cleaved LC3 exposing a C-terminal Gly120 (LC3-I) is further processed by ATG7 (E1-like), ATG3 (E2-like), and then conjugated to phosphatidylethanolamine (PE) with the help of, but is not essential, the ATG12-ATG5-ATG16L1 complex [60,61,62]. PE-conjugated LC3 (LC3-II) is localized onto both the inner and outer autophagosomal membranes, making it a useful autophagy marker by either analyzing the LC3 turnover (conversion of LC3-I to LC3-II) on immunoblotting or LC3 puncta formation under immunofluorescence confocal microscopy [63]. Interestingly, unlike yeast, mammals have four isoforms of ATG4 (ATG4A to ATG4D) and three LC3s (LC3A to LC3C). Moreover, the LC3/ATG8 family includes GABARAP proteins (GABARAP, GABARAPL1, and GABARAPL2/GATE-16), which appear to function in a later step of autophagosome maturation [64]. 

### 1.4. ATG9

A transmembrane protein ATG9-mediated cycling system containing the core proteins ATG9, ATG2, and WIPI1/2 (ATG18 in yeast) is also necessary for the elongation of the phagophore structure [65]. ATG9 is believed to deliver part of the membrane layers or phospholipid from donor sources to the expanding phagophore, although the precise mechanism remains to be addressed [66,67]. In yeast, the translocation of ATG9 into phagophore assembly site (PAS) depends on ATG11, ATG23, and ATG27 proteins, whereas the release of ATG9 from the phagophore elongation regions involves the ATG2-ATG18 and ATG1-ATG13 kinase complex. A sorting motif required for ATG9 trafficking has recently been identified [68]. This study further demonstrated that the mutations in this sorting motif resulted in the accumulation of ATG9 on the recycling endosomes and the blockage of autophagy, suggesting that the trafficking of ATG9 between the TGN and recycling endosomes is necessary for autophagy. Importantly, ATG9 trafficking appears to be regulated by the activities of two autophagy-initiating kinase complexes, ULK1 and PIK3C3/VPS34, and p38α MAPK/MAPK14 (mitogen-activated protein kinase 14) [66,69,70]. 

## 2. Autophagy Adaptors for Selective Autophagy

**Figure 2:**
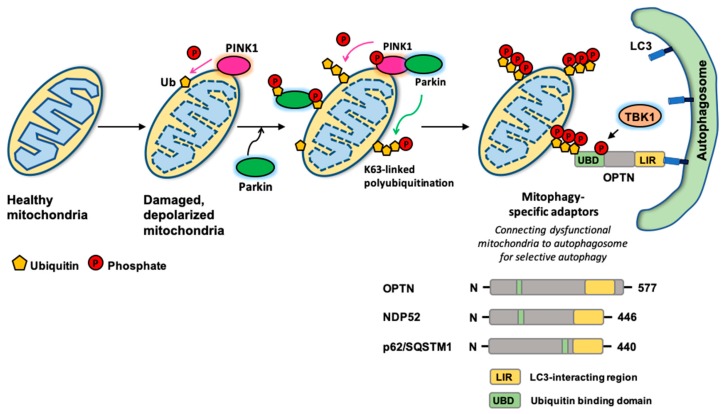
A schematic diagram of the selective autophagy for mitochondria, PINK1-Parkin dependent mitophagy. As a central place dictating cell survival and death, the clearance of damaged mitochondria by autophagy (mitophagy) is particularly important. Here, the PINK1-Parkin mediated ubiquitin-dependent pathway is introduced. In response to mitochondrial damage by depolarizing mitochondrial potential, PINK1 is no longer processed by a set of mitochondrial protease systems, and PINK1 is stabilized to accumulate on the mitochondria. And then, PINK1 recruits and activates Parkin by phosphorylating both ubiquitin and Parkin. Activated Parkin on mitochondria poly-ubiquitinates (mostly, Lys63-linked chain) myriad proteins on the damaged mitochondria. Mitophagy adaptors such as OPTN, NDP52, and p62/SQSTM1 function as a bridge between these poly-ubiquitin chains on the damaged mitochondria (via their UBD domain) and LC3 on the autophagosome (via their LIR motif). TBK1 is a protein kinase activated by mitochondrial damage, and phosphorylates the mitophagy adaptor OPTN to increase the binding affinity between OPTN and poly-ubiquitin chains on the dysfunctional mitochondria, thereby, accelerating mitophagy.

Autophagy has long been thought to be a non-selective bulk degradation pathway for cellular homeostasis in response to stressful conditions, especially for nutrient starvation. However, accumulating evidence has highlighted that selective autophagy accounts for the cellular quality control mechanism [71,72,73] against the protein inclusions from aggregate-prone or misfolded proteins (aggrephagy), dysfunctional or surplus organelles such as peroxisomes (pexophagy), mitochondria (mitophagy), ER (reticulophagy) and ribosomes (ribophagy), and pathogen infections (xenophagy). Among the various selective autophagy, much effort has been focused on mitophagy as mitochondria is a key factor for both cell survival (ATP synthesis) and death (release of pro-apoptotic factor, cytochrome c), and dysfunctional mitochondria is closely associated with various human pathophysiological conditions [74,75]. Additionally, the role of mitochondria in O_2_-coupled ATP synthesis sensitizes them to damage due to exposure to high levels of reactive oxygen species (ROS). The discovery of several autophagic adapters such as p62/SQSTM1, NBR1 (neighbor of *BRCA1* gene), OPTN (optineurin), NDP52 (nuclear domain 10 protein 52), and TAX1BP1 (TAX1 binding protein 1), has provided mechanistic insight into selective autophagy. In addition to these autophagy adaptors, it has become clear that modifications of the destructive cargo, especially ubiquitination, ensure the substrate recognition and specificity of autophagy adaptors [76]. A well-characterized mitophagy mechanism is based on the PINK1 ubiquitin kinase and Parkin E3 ubiquitin ligase (Figure 2) [75,77,78]. In normal conditions, PINK1 is imported into mitochondria to be cleaved and degraded by a set of mitochondrial protease systems such as the PARL protease. However, under the mitochondrial damage by the loss of mitochondrial membrane potential, PINK1 is no longer processed and stabilizes on the outer mitochondrial membrane. PINK1 then induces Parkin translocation and activation on the mitochondrial surface by phosphorylating both ubiquitin and Parkin at their respective Ser65 residues [79,80,81,82]. Activated Parkin adds massive Lys63-linked poly-ubiquitin chains on the damaged mitochondria. p62/SQSTM1, OPTN, NDP52, TAX1BP1, and NBR1 all have a ubiquitin-binding domain (UBD) to recognize these Lys63-linked poly-ubiquitinations on the damaged mitochondria and can recruit the LC3-decorated autophagosome via their LC3-interacting region (LIR) motif. Knockout of these five autophagy adaptors has determined that OPTN and NDP52 are required, yet redundant for mitophagy [83]. The ability of OPTN to compensate for NDP52 during mitophagy requires TBK1 (Tank-binding kinase 1), which is also necessary for xenophagy by phosphorylating OPTN at Ser177 to increase OPTN-LC3 binding [84]. For mitophagy, activated TBK1 in response to mitochondrial damage phosphorylates OPTN at Ser473 and Ser513 to increase the binding affinity between OPTN and poly-ubiquitin chains on the damaged mitochondria, resulting in the acceleration of mitophagy [85,86]. 

Outer mitochondrial membrane proteins Nix/BNIP3L (NIP3-like protein X), BNIP3 (BCL2 interacting protein 3), and FUNDC1 (FUN14 domain-containing protein 1) are other mitophagy receptors that fine-tune mitochondria populations in response to various stimuli. Nix/BNIP3L and BNIP3 are reported to play an important role in hypoxia-induced mitophagy [87,88]. Levels of BNIP3 and Nix/BNIP3L are transcriptionally up-regulated during hypoxia through hypoxia-inducible factor 1α (HIF-1α) [89]. BNIP3 interacts with LC3 and this interaction is promoted by BNIP3 phosphorylations at Ser17 and Ser24, which are located close to the LIR domain [90]. Both Nix/BNIP3L and BNIP3 also cross-talk with the PINK1-Parkin pathway [91,92]. Nix/BNIP3L ubiquitination by PINK1-Parkin promotes its interaction with LC3, enhancing mitophagy [93]. FUNDC1 is also a mitophagy receptor that promotes mitochondrial clearance in response to hypoxia [94,95]. CK2 phosphorylates FUNDC1 at Ser15 on the LIR motif, but, under hypoxic conditions, mitochondrial phosphatase PGAM5 dephosphorylates FUNDC1 to disrupt FUNDC1-mitochondria fusion regulator OPA1 interaction, thereby, inhibiting mitochondrial fusion. In turn, FUNDC1 translocates to ER-mitochondrial contact sites, recruiting the mitochondrial fission regulator DRP1 for mitochondrial fragmentation [96]. In addition, ULK1 interacts with and phosphorylates FUNDC1 at Ser17, which increases FUNDC1-LC3 interaction and stimulates mitophagy [97]. 

Recently, a mitochondrial inner membrane protein Prohibitin (PHB) has been reported to function as a mitophagy receptor for mitophagy [98,99]. In the Parkin-mediated mitophagy, mitochondrial membrane depolarization collapses the mitochondrial inner membrane to release PHB2 and subsequently associate with LC3, leading to phagophore formation at the proximity to the damaged mitochondria for mitophagy [98]. Similarly, the study from mitochondrial injury in cholestatic liver has shown that PHB2 forms a ternary complex with p62/SQSTM1 and LC3 to recruit autophagosomes for the clearance of damaged mitochondria [99]. Another putative mitophagy receptor is cardiolipin, a phospholipid within the mitochondrial inner membrane. Like PHB2, cardiolipin is also exposed to the cytosol when mitochondria is damaged, and interacts with LC3 to initiate the mitophagy program [100]. 

## 3. Post-Translational Modifications on Autophagy Proteins and the Regulation of Autophagy 

**Figure 3:**
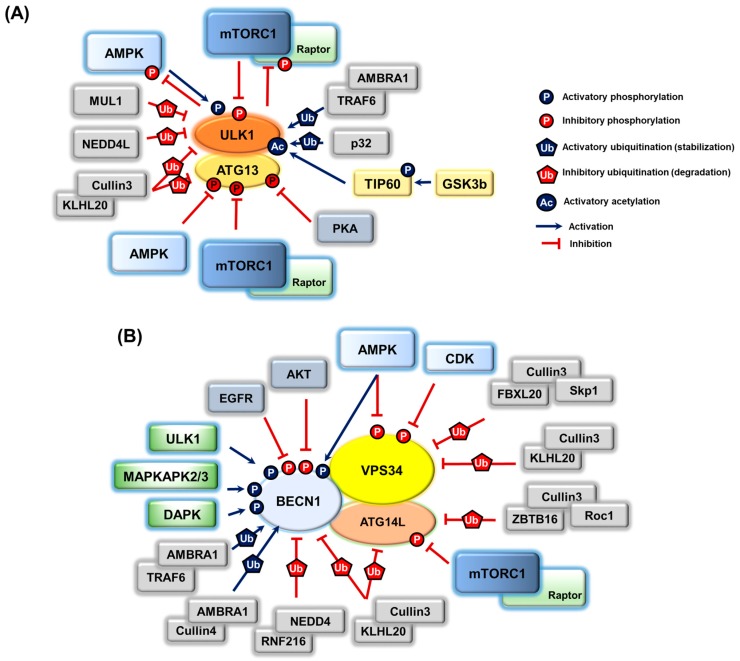
Post-translational modifications and regulations of two key autophagy-initiating kinase complexes, the ULK1 and PIK3C3/VPS34 complex. (**A**) The ULK1 complex is regulated by phosphorylation and is activated by multiple phosphorylations on a catalytic subunit ULK1 by AMPK, and inhibited by phosphorylation on ULK1 (mTORC) as well as ATG13 (mTORC1, PKA, and AMPK). Additionally, the ULK1 complex is activated by ubiquitination on ULK1 (AMBRA1-TRAF6 and p32), whereas it is negatively regulated by ubiquitination-dependent degradation (ULK1 by MUL1, NEDD4L, and Cullin3-KLHL20, ATG13 by Cullin3-KLHL20). In response to growth factor depletion, acetylation of ULK1 is increased by the activated GSK3-TIP60 acetyltransferase axis, resulting in autophagy induction. ULK1 may constitute a negative feedback loop to its upstream regulators, AMPK and mTORC1, by phosphorylating the mTORC1 subunit Raptor protein and all AMPK complex subunits. (**B**) Phosphoregulation of the PIK3C3/VPS34 complex is observed in the catalytic subunit VPS34 lipid kinase (AMPK and CDK for inhibition), BECN1 (AMPK, ULK1, MAPKAPK2/3, and DAPK for activation; AKT/PKB and EGFR for inhibition), ATG14L/Barkor (mTORC1 for inhibition), and UVRAG (mTORC1 for inhibition). Ubiquitinations on VPS34 (FBXL20-Skp1-Cullin1 and Cullin3-KLHL20 for degradation), BECN1 (NEDD4-RNF216 and Cullin3-KLHL20 for degradation, AMBRA1-TRAF6 or Cullin4 for stabilization), and ATG14L/Barkor (ZBTB16-Cullin3-Roc1 and Cullin3-KLHL20 for degradation) are also important for autophagy regulation.

As most upstream regulators of autophagy in autophagosome initiation and nucleation, both the ULK1 complex and pro-autophagy PIK3C3/VPS34 complex undergo a variety of post-translational modifications for their regulations (Figure 3).

### 3.1. ULK1 Complex

Accumulating biochemical analyses have shown that the ULK complex is extensively phosphorylated at multiple sites for regulation, where mTORC1 (mechanistic Target of Rapamycin Complex1) and AMPK (AMP-activated protein kinase) play a central role. Both mTORC1 and AMPK are key components in cellular energy homeostasis, especially for amino acid and glucose metabolism [101,102,103,104]. In addition to the place where autophagic cargoes are finally degraded, lysosome is an important platform for mTORC1 activation by the v-ATPase-Ragulator-Rag GTPase complex in response to amino acids [105,106,107,108]. Interestingly, the v-ATPase-Ragulator complex has also been recently shown to function as a lysosomal docking site for AXIN/LKB1-mediated AMPK activation [109], which in turn inactivates the Rag GTPase-Ragulator complex to suppress mTORC1 signaling under glucose starvation conditions [110]. 

mTORC1 inhibits the ULK1 complex by directly phosphorylating ULK1 at Ser638 and Ser758 [111,112], of which Ser758 phosphorylation is known to disrupt ULK1 activation by AMPK [112]. AMPK directly phosphorylates multiple residues on ULK1, which activates ULK1 to induce autophagy in response to glucose starvation [112,113]. mTORC1 has also been shown to directly phosphorylate ATG13 at Ser258, which suppresses ULK1 and negatively regulates autophagy induction [17]. Consistently, the TORC1-dependent phosphorylation of yeast ATG13 resulted in a dissociation of ATG13 from the ATG1 complex to inhibit ATG1 complex activity and autophagy [16]. Of note, the study documented that AMPK also phosphorylated ATG13 at Ser224 and this phosphorylation caused the inhibition of autophagy [17], representing a fine-tuning mechanism for the amplitude and duration of autophagy. Interestingly, there is a bidirectional regulation between AMPK/mTORC1 and ULK1. ULK1-dependent Raptor phosphorylation was reported to inhibit the interaction between mTORC1 and its substrate, and to suppress mTORC1 kinase activity [114,115]. Furthermore, ULK1 has been shown to add a negative feedback loop on the AMPK regulatory mechanism upon energy starvation, where ULK1 phosphorylates all three AMPK subunits to decrease AMPK activity [116]. The feedback regulatory mechanism between mTORC1, AMPK, and ULK1 may represent a signaling triad to fine-tune the energy/nutrient response. Similar to the mTORC1-dependent regulation of the ULK1 complex, lines of studies in yeast provided evidence demonstrating that cAMP-dependent protein kinase (PKA), a well-known metabolic signaling activating degradation of both glucose/glycogen and lipid, functions in autophagy [117,118]. Perplexingly, although PKA signaling is mostly activated upon nutrient starvation conditions, the data showed that inhibition of the PKA pathway resulted in the induction of autophagy, where PKA directly phosphorylated ATG13 at positions distinct from the TORC1 phosphorylation sites. PKA-dependent ATG13 phosphorylation blocked ATG1-ATG13 complex localization to the pre-autophagosomal structure in yeast [118]. This data suggests that the PKA and TORC1 pathways independently or cooperatively function to control autophagy in response to different nutrient sources.

In addition to phosphorylation, ubiquitination on ULK1 is becoming interested in ULK1 protein stability and kinase activity [119,120]. Under autophagy-inducing conditions, AMBRA1 recruits the E3-ubiqutin ligase TRAF6 that adds Lys63-linked, but not destructive Lys48-linked, poly-ubiquitin chains on ULK1, which promotes ULK1 dimerization and activation [119]. Notably, mTORC1 plays an important role in this Lys63-linked ULK1 ubiquitination process by directly phosphorylating AMBRA1 to disrupt AMBRA1-TRAF6 interaction. Considering that AMBRA1 is part of the PIK3C3/VPS34 complex and ULK1 functions as an upstream of the PIK3C3/VPS34 complex, the finding of AMBRA1-dependent ULK1 regulation may provide a positive feedback loop between two upstream autophagy regulators. Similarly, a chaperone-like protein p32 has been reported to function as a key regulator of ULK1 stability and the subsequent autophagy induction by forming a complex with ULK1 [120]. p32 depletion potentiated Lys48-linked, but impaired Lys63-linked, poly-ubiquitination of ULK1, leading to proteasome-mediated ULK1 degradation. p32-mediated Lys63-linked poly-ubiquitination on ULK1 promotes ULK1 stability, which is required for both starvation-induced autophagy and mitophagy. In another context, ubiquitination negatively regulates ULK1 signaling by degrading ULK1 [121,122,123]. Upon starvation, the other E3 ubiquitin ligases, MUL1, NEDD4L, and Cullin3-KLHL20, control the amplitude and duration of the autophagic response by driving ULK1 degradation via Lys27/29-linked (NEDD4L) [122] or Lys48-linked (MUL1 and Cullin3) poly-ubiquitination [121,123]. 

Accumulating evidence has shown the importance of acetylation on the regulation of ULK1 and autophagy. First, a mammalian acetyltransferases TIP60 has been reported to activate the ULK1 complex [124]. Under growth factor, but not glucose, starvation, the activated glycogen synthase kinase-3 (GSK3) by inactivation of its negative regulatory PI3K-AKT/PKB signaling stimulates the acetyltransferase activity of TIP60 by directly phosphorylating TIP60 at Ser86. TIP60 acetylates and activates ULK1 kinase for autophagy induction. A recent report provided a similar result where, upon ER stress, GSK3 is activated and, subsequently GSK3-dependent TIP60 phosphorylation is elevated, which triggers ULK1 acetylation and autophagy induction [125]. 

### 3.2. PIK3C3/VPS34 Complex

A number of phosphorylations and their regulations have also been shown in the PIK3C3/VPS34 complex and its regulatory proteins. First, similar to ULK1 phosphoregulation by the AMPK and mTORC1 pathways, VPS34, BECN1, ATG14L/Barkor, and UVRAG are also directly phosphorylated and regulated by these nutrient sensing signaling pathways. AMPK is able to phosphorylate VPS34 for the inhibition of the non-autophagic PIK3C3/VPS34 complex, whereas it phosphorylates BECN1 for the activation of the pro-PIK3C3/VPS34 complexes such as ATG14L-containing PI3KC3-C1 and UVRAG-containing PIK3C3-C2 [126]. Importantly, this study showed that the preference of VPS34 (Thr163/Ser165) and BECN1 (Ser91/Ser94, which is equivalent to S93/S96 in humans) phosphorylation by AMPK was determined by ATG14L/Barkor. In the presence of ATG14L/Barkor, inhibitory phosphorylation on VPS34 was suppressed, but the phosphorylation of Beclin1 was greatly enhanced to activate the complex. mTORC1 directly phosphorylates ATG14L/Barkor at multiple residues to inhibit the PIK3C3/VPS34 complex [127]. Accordingly, this negative regulation was not observed in ATG14L-free PIK3C3/VPS34 complexes. Similarly, mTORC1 also phosphorylates UVRAG to inhibit the complex by recruiting the inhibitor protein RUBICON into the UVRAG-associated complex [36]. Upon amino acid starvation, which blunts mTORC1 signaling, mTORC1-dependent inhibitory UVRAG phosphorylation is diminished to release UVRAG from RUBICON, allowing UVRAG-HOPS complex interaction for autophagosome maturation with lysosome. 

VPS34 is also a target of CDKs (cyclin-dependent protein kinases), indicating the cross-talks between autophagy and the cell cycle [128]. VPS34 is phosphorylated at Thr159 by CDK1, which negatively regulates its interaction with BECN1 to inhibit autophagy during mitosis. Additionally, a neuronal CDK5/p25 is capable of phosphorylating VPS34 at Thr159 [128]. Considering that the specific functions of CDK5 in the nervous system [129] and in Alzheimer’s disease by tau phosphorylation [130], CDK5-dependent VPS34 phosphorylation and the following inhibition of the complex may underlie the molecular basis of neurodegeneration. 

Among the various subunits on the PIK3C3/VPS34 complex, BECN1 is a protein that is extensively phosphorylated by many different kinases, of which AMPK, ULK1, MAPKAPK2/3 (mitogen-activated protein kinase-activated protein kinase 2 and 3), and DAPK (death-associated protein kinase) promote autophagy activation, whereas AKT/PKB and EGFR (epidermal growth factor receptor) inhibit autophagy. Therefore, BECN1 is a platform that integrates multiple signaling pathways in which these kinases are involved. ULK1 activates the PI3KC3-C1 complex by phosphorylating BECN1 at Ser14 (Ser15 in humans) [131]. Similar to the activation of the ATG14L-containing PI3KC3-C1 complex by AMPK, interaction between ULK1 and BECN1 was enhanced by ATG14L/Barkor, which was reflected by an increase of BECN1 phosphorylation. Interestingly, the ability of ATG14L/Barkor to promote BECN1 phosphorylation was abolished in the ATG14L/Barkor mutants defective in phagophore targeting, indicating that the activation of the ATG14L-containing PI3KC3-C1 complex by ULK1 may occur at the phagophore. Two members of the p38 mitogen-activated protein kinase (MAPK) signaling pathway, MAPKAPK2 (MK2) and MAPKAPK3 (MK3), also directly phosphorylate BECN1 at Ser90, leading to autophagy [132]. Interestingly, MK2/MK3-dependent BECN1 phosphorylation is blocked by Bcl-2, a negative regulator of BECN1, thereby, MK2/3-dependent BECN1 phosphorylation may participate in the regulatory mechanism by which Bcl-2 inhibits the autophagy function of BECN1. Similarly, recent data have supported the significance of BECN1 Ser90 phosphorylation in autophagy [133]. This study identified that protein phosphatase 2A (PP2A) and DAPK3 were involved in the BECN1 Ser90 phosphorylation cycle, which is important for autophagy induction. Furthermore, it has also been reported that mTORC1-dependent inhibitory phosphorylations on ULK1 are blunted by PP2A in response to the nutrient starvation [134]. Under nutrient-rich conditions, PP2A is inactivated by binding to an inhibitory protein α4, but, as nutrients are depleted, PP2A is released from this latent complex, resulting in rapid dephosphorylation of ULK1, followed by autophagy induction. Interestingly, this study showed that PP2A activity is abnormally high in pancreatic ductal adenocarcinoma cells that require high basal autophagy for viability, proposing the novel mechanism by which the phosphatase activity toward ULK1 allows cancer cells to maintain high autophagy activity even in the presence of active mTORC1 signaling for high growth and survival capability in cancers. Besides BECN1 Ser90 phosphorylation, DAPK also phosphorylates BECN1 at Thr119, which is placed onto the BH3 domain that is required for Bcl-2 binding to BECN1. In this sense, DAPK-dependent BECN1 Thr119 phosphorylation may represent another layer of the autophagy activating mechanism by reducing the interaction of BECN1 with its inhibitor Bcl-2 [135].

In contrast to the activating phosphorylations above-mentioned, multiple EGFR-dependent BECN1 tyrosine phosphorylations (Tyr229, Tyr233, and Tyr352) have been reported to suppress autophagy [136]. They increase BECN1-Bcl-2 binding, and decrease BECN1-associated VPS34 kinase activity. Importantly, in non-small-cell lung carcinoma (NSCLC) tumor xenografts, the expression of a EGFR-dependent tyrosine phosphorylation mimetic BECN1 mutant resulted in reduced autophagy activity, increase of tumor growth, and resistance to TKI (tyrosine kinase inhibitor) therapy. A well-known oncogenic protein kinase AKT/PKB also negatively regulates autophagy by phosphorylating BECN1 at Ser295 [137]. Similarly, the expression of the BECN1 mutant incapable of AKT/PKB-dependent phosphorylation results in the increase of autophagy, and the decrease of anchorage-independent growth as well as tumorigenesis. 

In addition to phosphoregulations on the PIK3C3/VPS34 complex proteins, ubiquitination represents another layer of the regulatory mechanism. A genome-wide RNAi screening identified a F-box protein, FBXL20, as inhibiting autophagy flux and decreasing cellular PI(3)P level [138]. A subsequent study further demonstrated that upon DNA damage, CDK1-dependent VPS34 Thr159 phosphorylation promoted its ubiquitination and proteasomal degradation mediated by FBXL20 and the associated Skp1 (S-phase kinase-associated protein-1)-Cullin1 complex, leading to the inhibition of autophagy and receptor endocytosis [139]. Moreover, ubiquitination and degradation of ATG14L/Barkor has been shown to be under the control of the ZBTB16-Cullin3-Roc1 E3 ubiquitin ligase complex [140]. BECN1 undergoes Lys11/48-linked poly-ubiquitination by NEDD4 and RNF216 ligases, respectively, leading to its degradation and autophagy inhibition [141,142]. Consistent with the negative regulation of BECN1 by ubiquitinations, deubiquitinases USP10/USP13 have been reported to stabilize BECN1 and activate the BECN1-containing PIK3C3/VPS34 complex [143]. Interestingly, BECN1-interacting AMBRA1 also functions in BECN1 ubiquitination. Notably, AMBRA1-mediated BECN1 ubiquitination is not a target for degradation and inhibition, but is involved in the stabilization of the BECN1-containing PIK3C3/VPS34 complex and the activation of autophagy. The AMBRA1-Cullin4 ligase or AMBRA1-TRAF6 ligase complex confer the Lys63-linked poly-ubiquitination of BECN1, promoting autophagy [144,145]. However, AMBRA1 is transiently dissociated from the Cullin4 ligase in the early stage of autophagy in a ULK1-dependent manner. The releasing AMBRA1 inhibits the Cullin5 ligase that activates mTORC1 signaling by degrading a mTOR inhibitor protein DEPTOR, thereby establishing a positive feedback loop to facilitate a rapid induction of autophagy [146]. The Cullin3-KLHL20 ubiquitin ligase complex also targets a set of autophagy machinery proteins such as ULK1, ATG13, VPS34, BECN1, and ATG14L for proteasomal degradation [123]. Considering the ubiquitination and degradation of both the ULK1 and PIK3C3/VPS34 complex by Cullin3-KLHL20, this ubiquitination system may represent the fine-tuning mechanism for the duration and amplitude of the autophagy response.

### 3.3. Regulation of Other Autophagy Machinery Proteins by Post-Translational Modifications

Accumulating efforts have demonstrated that many ATG proteins are post-translationally modified and regulated in response to a variety of stimuli [65,147]. First, phosphoregulation has been observed in LC3, p62/SQSTM1, and ATG9. PKA directly phosphorylates LC3 at Ser12, and inhibits LC3 lipidation (LC3-II) and LC3 binding on the autophagosomal membrane, resulting in the inhibition of autophagosome formation [148]. Similarly, PKC also directly phosphorylates LC3 at Thr6 and Thr29 in vitro, but these phosphorylations are not the molecular basis for the inhibitory effects of PKC on autophagy [149]. GABARAP and GABARAPL1, a member of the LC3/ATG8 family for autophagosome biogenesis, are also able to be phosphorylated by the MAPK15/ERK8 kinase (Mitogen-Activated Protein Kinase 15), which induces their lipidation, resulting in autophagosome formation and p62/SQSTM1 degradation [150]. Interestingly, upon starvation, activated MAPK15 moves into the autophagosomes and prevents the inhibitory phosphorylation of LC3 by PKA [150]. PKA-dependent p62/SQSTM1 phosphorylation at the N-terminal Phox and Bem1p (PB1) domain required for atypical protein kinase C (aPKC) interaction and p62 oligomerization disrupts the interaction of p62/SQSTM1 with these interacting partners [151]. However, the relationship between PKA signaling and autophagy should be more carefully examined because most of the evidence has pointed out the inhibitory effects of PKA on autophagy, although PKA is generally activated in autophagy-inducing conditions, for example, metabolic stresses. p38 MAP kinase phosphorylates ATG5 at Thr75 and inhibits autophagy [152]. Upon autophagy-inducing conditions, the growth arrest and DNA damage of the 45 beta (Gadd45β)-MAPK/ERK kinase kinase 4 (MEKK4) pathway specifically directs p38 MAP kinase to the autophagosome, which allows p38 MAP kinase to phosphorylate and inhibit ATG5, thereby blocking the autophagy process to accumulate autophagosomes. In contrast, ATG1-dependent ATG9 phosphorylation in yeast has been shown to recruit ATG8 and WIPI-1/ATG18 to the phagophore assembly site, activating autophagosome formation [153]. 

Ubiquitination plays an important role in the regulation of ATG5, ATG7, ATG8, and ATG12 [154]. Under nutrient-rich conditions, the acetyltransferase p300 was reported to directly interact with ATG7 and, concomitantly, acetylates ATG5, ATG7, ATG8, and ATG12 [155]. However, during starvation, p300 is dissociated from ATG7, allowing SIRT1 to deacetylase these autophagy machinery proteins, leading to autophagy induction [156]. In contrast, Esa1 (a yeast orthologue of TIP60)-dependent Atg3 (ATG3) acetylation at Lys19 and Lys48 is reported to be required for autophagy by increasing ATG3 and ATG8 (LC3 in mammals) interaction, followed by the lipidation of ATG8 (LC3-II in mammals) [157]. 

Finally, specific cleavages of autophagy proteins by calpains (CAPNs) and caspases (CASPs) have been widely reported to balance and switch autophagy/apoptosis in response to various stimuli. ATG5 is capable of being cleaved at Thr193 by CAPNs, but not CASPs, and the resulting N-terminal ATG5 fragment is no longer an autophagy machinery protein [158]. Instead, it can induce apoptosis through binding to Bcl-xL at the mitochondria and promoting the release of cytochrome c as well as the activation of CASPs [158]. Interestingly, CASPs-dependent ATG5 cleavage has also been observed in several melanoma cell lines by the combination of tumor necrosis factor-related apoptosis-inducing ligand (TRAIL) and arginine deprivation, which contributes to apoptosis [159]. BECN1 is another example of the cleavage-dependent autophagy–apoptosis switch. Many CASPs such as CASP3, CASP6, CASP8, CASP9, and CASP10 have been reported to cleave the C-terminus of BECN1 at different sites: Asp133 [160,161], Asp146 [161], and Asp149 [160,162]. The resulting C-terminal BECN1 fragment cannot activate autophagy, but induces apoptosis by translocating into the mitochondria and, then cooperating with pro-apoptotic BCL2 members. Moreover, the cleavage of ATG3 at Asp169 by CASP8 [163] and the cleavage of ATG16L1 at Thr300 by CASP3 [164] impairs autophagy during receptor-activated cell death or bacterial infection. In contrast, the cleavage of ATG4 determines a substrate specificity in the LC3 turnover process [165]. CASP3-dependent cleavage of ATG4D at Asp163 specifically enhances the priming and delipidation of GABARAPL1, but not the other LC3 family members, GABARAPL2 and LC3, in human cancer cells.

## 4. Transcriptional Regulation of Autophagy 

Accumulating evidence has indicated that autophagy regulation at its transcriptional level is also important for autophagic responses to specific stimuli [166,167], where transcription factor EB (TFEB) is known as a master transcriptional regulator of autophagy [168]. TFEB binds to the CLEAR (Coordinated Lysosomal Expression And Regulation) element on a variety of autophagy and lysosomal genes to induce their expression, which increases both autophagosome and lysosome biogenesis, and promotes autophagosome–lysosome fusion [169]. Transcriptional activity of TFEB is tightly regulated by phosphorylation. Under nutrient-rich conditions, ERK2, AKT/PKB, and mTORC1 have been shown to inhibit TFEB by sequestering in the cytoplasm [169,170,171]. In contrast, under the starvation conditions that blunt mTORC1 signaling, inhibitory phosphorylations on TFEB are diminished and TFEB translocates to the nucleus. Interestingly, recent evidence has demonstrated another layer of TFEB regulation by AMPK [172,173]. Once AMPK is activated by glucose starvation, it phosphorylates the FOXO3 transcription factor in the nucleus, which in turn transcriptionally represses E3 ubiquitin ligase SKP2 (S-phase Kinase-associated Protein 2) expression. This suppression of the SKP2 protein level results in the stabilization and increase of CARM1 (Coactivator-Associated arginine Methyltransferase 1) to interact with TFEB and activate TFEB-dependent transcription by modifying the histone structure via Histone3 Arg17 dimethylation [172]. In addition to the FOXO3-SKP2-CARM1 axis, AMPK also participates in TFEB regulation by phosphorylation of acetyl-CoA synthase 2 (ACSS2). AMPK-dependent ACSS2 phosphorylation at Ser659 induces its nuclear translocation by binding to importin α5 to interact with TFEB, resulting in the spatiotemporal increase of acetyl donor acetyl-CoA to promote Histone3 acetylation at TFEB target gene promoters for transcriptional activation [173]. The two arms of TFEB regulation by AMPK and mTORC1 may represent a fine-tuning transcriptional regulation mechanism by which mTORC1 inactivation and AMPK activation cooperatively induce autophagy and lysosome genes by altering TFEB localization and the transcription allowance of the target genes via chromatin remodeling, respectively. Another transcription factor ZKSCAN3 (Zinc finger with KRAB and SCAN domains 3) has also been shown to play a role in this transcriptional regulation [174], where ZKSCAN3 functions as a transcriptional repressor of the autophagy–lysosome pathway. Nutrient deprivation leads to the nuclear export of ZKSCAN3 and releases transcriptional repression of autophagy gene expression. Notably, a recent study has demonstrated that PKC (especially α and δ isoforms) couples the activation of the TFEB transcription factor with the inactivation of the ZKSCAN3 transcriptional repressor through two separate signaling pathways in parallel with mTORC1 [175]. Once PKC is activated, it phosphorylates and inhibits GSK3β, resulting in the decrease of inhibitory phosphorylation, nuclear translocation, and activation of TFEB. Additionally, PKC activates JNK and p38 MAPK to phosphorylate ZKSCAN3, leading to its inactivation by translocating to the cytoplasm. 

## 5. The Roles of Autophagy in Human Pathophysiological Conditions 

### 5.1. Metabolism

Nutrient starvation induces autophagy to recycle the essential metabolites to maintain cell viability [4,176,177]. At first, autophagy is under the tight control of the cellular amino acid level. In response to amino acid starvation, autophagy replenishes the cellular amino acid level by degrading surplus cellular contents, which are used to synthesize the proteins required for survival as well as to feed into key metabolism such as pyruvate, TCA cycle intermediates, and acetyl-CoA. Growing evidence has indicated the importance of autophagy in lipid metabolism [178,179,180]. Selective degradation of lipid droplets by autophagy, lipophagy, produces free fatty acids from triglycerides [178]. It has been reported that a hepatic ATG7 knockout mouse showed malfunctions in lipid metabolism as evidenced by increases in both the number and size of the lipid droplets, and eventually in the increase in liver size. However, in contrast to the ability of lipid breakdown, autophagy is also involved in the formation of white adipose tissue [181,182]. Inhibition of autophagy blocks white adipocyte differentiation, and adipose-specific ATG7 knockout results in lean mice with decreased white adipose mass and enhanced insulin sensitivity. Notably, the white adipocytes in these mice showed some key features of brown adipose tissue, for example, an elevated rate of fatty acid β-oxidation. This suggests that autophagy functions to regulate body lipid accumulation by controlling adipocyte differentiation and determining the balance between white and brown fat. 

The importance of autophagy in metabolic regulation has been clearly demonstrated in several autophagy-defective mouse models [183,184]. The autophagy-defective mice such as ATG5 or ATG7 knockout mice were born without any notable defects, but died soon after birth. Analysis of key metabolites in these mice showed systemic amino acid deficiency and severe hypoglycemia. Hypoglycemia is normally observed in newborn mice because maternal glucose supply is terminated upon delivery, but glucose level is normally increased within a few hours after delivery. During this time window, the metabolic pathways for gluconeogenesis are not yet fully established. Instead, autophagy-dependent glycogen breakdown, glycophagy, in the liver plays an important role in the systematic glucose homeostasis [185,186,187]. Apart from the cytosolic glycogen degradation by glycogen phosphorylase cascade, newborn mice have an additional glucose supply system in the lysosome [188,189], where lysosomal glycogen hydrolyzing enzymes including glucan 1,4-α-glucosidase degrade glycogen to provide non-phosphorylated glucose during the neonatal starvation period [190]. Recent studies have suggested that glycophagy is not limited to the neonates, but also implicated in some muscle disorders [2]. For instance, accumulation of glycogen granules in the muscles of patients with Danon disease, which causes cardiomyopathy, proximal muscle weakness, and mental retardation [191], is demonstrated primarily by the defects in autophagy. Defective autophagy also seems to be a cause of the accumulation of polyglucosans, poorly branched glycogen bodies, in organs from patients with Lafora disease [192]. In addition to glycogen breakdown, autophagy appears to contribute to glucose metabolism by modulating pancreatic β-cell function [193,194]. Autophagy-defective mice specifically in the β-cell reveal progressive β-cell degeneration and decreased insulin secretion.

### 5.2. Tumorigenesis

Autophagy also plays a crucial role in tumorigenesis as well as in the sensitivity against cancer chemotherapeutics [195,196,197,198]. First, it has been extensively demonstrated that autophagy has a tumor-suppressive role as a cellular homeostasis program. A number of studies have documented the changes of many *ATG* genes such as ATG2, ATG5, ATG9, ATG12, and UVRAG in various cancers [199,200]. Importantly, *BECN1* is well-known to be mono-allelically deleted in around 50% of breast, ovarian, and prostate cancers [201,202]. Furthermore, experimental mouse models have shown a high incidence of lymphomas, liver, and lung cancers in *BECN1 +/-* heterozygote mice [203,204]. Similarly, it has been reported that the protein level of Bif-1, an autophagy effecter involved in autophagosome biogenesis as part of the PIK3C3/VPS34 complex, was decreased in gastric and prostate cancers, and Bif-1 knockout mice are prone to tumorigenesis [205]. Dysregulation of autophagy is very likely to accumulate reactive oxygen species (ROS) and a concomitant increase of DNA damage such as double-strand breaks and polyploid nuclei [206,207]. Additionally, a positive correlation between tumor progression and autophagy adaptor p62/SQSTM1 protein level was documented in vivo [208]. Accumulation of p62/SQSTM1 induced by the inhibition of autophagy has been shown to increase endoplasmic reticulum (ER) stress, DNA damage, and induce the deregulation of the nuclear factor kappa B (NF-kB) and antioxidant nuclear factor erythroid 2-related factor 2 (NRF2) pathways in many cancer cells [209,210].

Autophagy also participates in the oncogenic process. Cancers are always exposed to stressful conditions, especially metabolic stresses due to the limited nutrient and oxygen supplies where autophagy protects cancer cells by fueling the energy sources [176,211]. A recent metabolome analysis has demonstrated that hypoxia increases the catabolic intermediates of proteins and lipids to maintain minimal levels of mitochondrial ATP production [212]. Notably, the inhibition of autophagy in this experimental setting decreases intracellular ATP level and induces cell death. In line with this notion, recent efforts have demonstrated that metabolic coupling between cancer cells and their stroma cells is closely related to autophagy [213,214]. It has been shown that tumor stromal fibroblasts show increased autophagy activity and the metabolites from these fibroblasts such as lactate, ketone bodies, and amino acids are used to fuel energy production and cancer growth. In addition, the importance of autophagy in many aspects of oncogenesis such as growth/proliferation, resistance to cell death, and metastasis has been observed in many cancer models driven by oncogenic *RAS* [215,216,217,218,219]. Consistently, the inhibition of autophagy in several different cell lines harboring oncogenic *RAS* mutations was shown to blunt anchorage-independent cell growth [220].

### 5.3. Neurodegeneration

As an important intracellular quality control system removing damaged/long-lived organelles and protein aggregates, the growing body of evidence has indicated the significance of autophagy in several neurodegenerative diseases. The findings of the accumulation of ubiquitin-positive inclusion bodies in neurons from the neural cell-specific autophagy-defective mice [20,21], which also develop progressive motor deficits and abnormal reflexes, suggest that autophagy may play important roles in neuro-pathophysiological conditions accumulating toxic protein aggregates such as extended polyglutamine-containing proteins in Huntington’s disease, and mutant forms of α-synuclein in familial Parkinson’s disease [221,222]. CMA also participates in physiological α-synuclein turnover, but mutant forms of α-synuclein become resistant to CMA [223,224].

Dysregulation of autophagy has also been observed in Alzheimer’s disease, but the contribution of autophagy appears to be different to other types of neurodegeneration. Of note, autophagosome-like structures accumulate in dystrophic neurites of Alzheimer’s disease patients and model mice [225]. Very surprisingly, the protease responsible for the cleavage of the Aβ precursor protein, APP, is found in these autophagosome-like compartments. This study suggests that accumulation of autophagosomes by inhibition of autophagic flux may provide an alternative route for neurotoxic Aβ peptide production in Alzheimer’s disease [225].

## 6. Closing Remarks

Since a Belgian biochemist, Dr. Christian de Duve, a Nobel Prize laureate in 1974, first discovered and described “autophagy”, which literally means “self-eating”, significant progress has been made toward understanding the molecular basis of the autophagy process and regulation, of which remarkable contributions to the identification of essential autophagy genes provided a Japanese scientist, Yoshinori Ohsumi, with another Nobel Prize in Physiology or Medicine in 2016. One of the long-standing questions in studies of autophagy is how autophagy responds to a variety of environmental and intracellular stimuli for cellular homeostasis. Accumulating reports demonstrating the role of both basal and enhanced autophagy activity in many pathophysiological conditions have indicated multiple layers of the regulatory mechanism corresponding to the distinct cellular context. Furthermore, the increasing concern in selective autophagy has highlighted that autophagy is an essential recycling as well as quality control program in metabolism, the turnover of damaged/long-lived organelles, clearance of toxic threats, and pathogen infections. However, many questions still exist in the autophagy field. Notably, recent reports have identified some “noncanonical” autophagy that does not rely on or bypasses the essential autophagy proteins [226]. Another key question is how autophagy is differently regulated in response to cell- or tissue-type specificity. In addition, studies of autophagy in the cultured cell lines should always be carefully examined as there is a concern that the artificial stress conditions might fail to adequately mimic the true physiological conditions. Considering the physiological significance of autophagy in many human diseases, therefore, the establishment of a reliable tool to study autophagy in vivo will be of great interest to obtain insights into autophagy as a novel therapeutic target. In this sense, the development of a method to monitor the autophagy activity in vivo and of pharmacological drugs specifically targeting autophagy will provide an opportunity to obtain important clues for these unsolved questions.
